# Primary Cutaneous CD30-Positive Lymphoproliferative Disorders—Current Therapeutic Approaches with a Focus on Brentuximab Vedotin

**DOI:** 10.3390/jcm13030823

**Published:** 2024-01-31

**Authors:** Tomasz Stein, Tadeusz Robak, Wojciech Biernat, Ewa Robak

**Affiliations:** 1 Department of Dermatology, Medical University of Lodz, 90-647 Lodz, Poland; t.stein@wp.pl; 2 Department of Hematology, Medical University of Lodz, 93-510 Lodz, Poland; 3 Department of General Hematology, Copernicus Memorial Hospital, 93-510 Lodz, Poland; 4 Department of Pathomorphology, Medical University of Gdansk, 80-214 Gdansk, Poland; wojciech.biernat@gumed.edu.pl

**Keywords:** anaplastic large cell lymphoma, brentuximab vedotin, CD30-positive lymphomas, diagnosis, lymphomatoid papulosis, primary cutaneous anaplastic large cell lymphoma, treatment

## Abstract

One of the most common subgroups of cutaneous T-cell lymphomas is that of primary cutaneous CD30-positive lymphoproliferative disorders. The group includes lymphomatoid papulosis (LyP) and primary cutaneous anaplastic large cell lymphoma (pcALCL), as well as some borderline cases. Recently, significant progress has been made in understanding the genetics and treatment of these disorders. This review article summarises the clinical evidence supporting the current treatment options for these diseases. Recent years have seen the introduction of novel agents into clinical practice; most of these target CD30, such as anti-CD30 monoclonal antibodies and conjugated antibodies (brentuximab vedotin), bispecific antibodies and cellular therapies, particularly anti-CD30 CAR-T cells. This paper briefly reviews the biology of CD30 that makes it a good therapeutic target and describes the anti-CD30 therapies that have emerged to date.

## 1. Introduction

Primary cutaneous CD30+ lymphoproliferative disorders (LPDs) are the second most common group of primary cutaneous T-cell lymphomas, after mycosis fungoides (MFs) [1]. The group includes primary cutaneous anaplastic large cell lymphoma (pcALCL), lymphomatoid papulosis (LyP) and borderline tumours. They generally have a good prognosis, but most cases are also characterised by a long-term and recurrent course that makes everyday life difficult for patients. It is worth emphasising that although these are indolent proliferations, they may pose a risk of internal organ involvement. In addition, although LyP is a benign disease, it is still included in the group of lymphomas because, in rare cases, it can transform from LyP to MF, pcALCL or Hodgkin lymphoma (HL). Generally, LPD30+ disorders have a slow and chronic course. Currently, brentuximab vedotin (BV) is one of the newer drugs used to treat CD30+ LPD [2]. Brentuximab vedotin is a conjugate of a mouse–human chimeric IgG1 anti-CD30 antibody linked to monomethyl auristatin E (MMAE), an anti-tubulin drug. Treatment brings the rapid remission of skin lesions but also causes side effects, the most common of which is peripheral neuropathy. It is believed that the neuropathy that can develop during brentuximab vedotin therapy is caused by the inhibition of microtubule-dependent axonal transport.

CD30, also known as TNFRSF8 (TNF Receptor Superfamily Member 8) or Ki-1, is a 120 kDa glycoprotein receptor belonging to the TNF family [3]. It was first identified in 1982 as a surface marker for selected Hodgkin lymphoma (HL) cell lines [4]. The CD30 receptor is activated by the binding of its ligand, CD30L, a membrane-bound cytokine expressed on activated granulocytes and lymphocytes [5]. CD30 is most highly expressed in CD8+ and CD4+ lymphocytes [6]. The formation of the ligand complex activates the receptor and TNF-related factor recruitment (TRAF1, TRAF2, and TRAF3); it also initiates the binding of proteins to form a signalling complex inside the cell. This stimulates the nuclear factor kappa B (NFkB) pathway and activates signalling via the mitogen-activated protein kinase (MAPK) pathways. Both pathways promote a variety of effects, including those that promote survival and prevent apoptosis of neoplastic cells. Moreover, it is postulated that CD30 activation is regulated via the expression of Jun-B, which is a transcription factor of activated proteins (AP-1) responsible for neoplastic transformation [7].

In healthy individuals, CD30+ expression is minimal, most prominent in activated lymphocytes; even in this case, it only accounts for less than 1% of activated lymphocytes. In healthy individuals, the role of CD30 is thought to supervise the immune system by mediating information between B and T cells.

Importantly, CD30 expression is also observed on CD8+ and CD4+ lymphocytes in skin inflammation, viral infections and malignancies. Increased amounts of CD30 have been found in atopic dermatitis, psoriasis, parasitic infections (scabies) [8], as well as in viral infections with Epstein–Barr virus (EBV), human immunodeficiency virus (HIV), human T-lymphoma virus 1 (HTLV-1) and molluscum contagiosum [9]. Falini et al. showed that the viral infection could increase the number of activated CD30-expressing cells from 0.1% to 95% within three days [10].

CD30 expression is characteristic of LyP and pcALCL; however, it is not unique to primary cutaneous CD30+ LPD and cannot be defined as a marker of these disorders. It is also found in anaplastic large cell lymphoma (ALCL), HL, large cell transformation of mycosis fungoides (MF-LCT), acute myeloid leukaemia (AML), myelodysplastic syndromes (MDS), mastocytosis, CD30+ B-cell lymphomas and EBV+ hydroa vacciniforme-like-T-cell lymphoma [11]. CD30 expression varies across the six WHO-recognised LyP histological subtypes. Primary cutaneous anaplastic large cell lymphoma cells express CD30 at 75%.

In clinical practice, CD30 expression on the cell surface can be assessed by flow cytometry (FCM) and immunohistochemistry (IHC) [9]. The most common evaluation method is the immunohistochemistry of skin specimens, which can be combined with an assessment of cell morphology. There are many different anti-CD30 antibodies that recognise epitopes on CD30, but the BerH2 antibody is used for the routine IHC assessment of CD30 [5]. Given recent molecular discoveries, therapies targeting the CD30 receptor appear attractive to researchers trying to identify targeted therapies for these lymphomas.

## 2. Primary Cutaneous CD30-Positive Lymphoproliferative Disorders

According to the current consensus of the fifth edition of the classification of cutaneous lymphomas of the World Health Organization (WHO) and the European Organization for Research and Treatment of Cancer (EORTC), primary cutaneous CD30+ LPD constitutes a separate group of lymphomas [1,12]. They are the second most common group of CTCLs, accounting for approximately 25% [13,14].

Primary cutaneous CD30+ LPDs include pcALCL and LyP, as well as borderline tumours with clinical and histological features lying between the two. Importantly, as the histological criteria are often not sufficient to distinguish between these diseases, researchers typically use the term CD30+ LPD during the initial clinical evaluation, especially pathological diagnosis, rather than LyP or pcALCL. A short follow-up period (8–10 weeks) may reveal spontaneous regression, which is more characteristic of LyP. This diagnosis is critical for further disease management and the initiation of appropriate therapy. Both LyP and pcALCL have different histological, clinical and immunophenotypic variants. Also, despite their histological picture suggesting highly malignant neoplastic infiltration, they also generally have a slow course with a good prognosis [15].

### 2.1. Lymphomatoid Papulosis

Lymphomatoid papulosis was first described by Dr. Warren L. Macauley in 1968 as a benign, histologically malignant, self-limiting, but recurrent disorder of unknown aetiology [16]. It was only after years of follow-up that biopsies from skin lesions demonstrated a histology typical of lymphoma together with the presence of large atypical CD30-positive cells; as such, LyP was classified as a CD30+ LPD [17].

Lymphomatoid papulosis is a rare skin proliferation with an incidence of 1.2–1.9/million and an excellent prognosis, with a 10-year survival rate of approximately 100% [8]. However, patients with LyP are at risk of developing secondary malignancies, including nodal or cutaneous ALCL, HL and MF. These lymphomas are clonally related to LyP and, according to various sources, develop in 4 to 60% of LyP patients. They can occur before, concomitantly or after LyP [8,18,19]. The most common secondary lymphomas identified in a large retrospective cohort study of 180 patients with LyP were MF (61.4%) and ALCL (26.3%) [20]. Sauder et al. report that LyP B or C, male sex, LyP with monoclonal rearrangement of the TCR receptor, EBV infection and advanced age increase the chance of second malignancy; as such, each patient diagnosed with this disease requires increased oncological supervision [13].

The pathogenesis of LyP is unknown, but most studies suggest a genetic background based on abnormalities in the CD30 transcription [21]. Although viral infections with HTLV-1 virus and hepatitis E virus were also suspected, these correlations were not confirmed in subsequent studies [22,23]. It has been suggested that LyP may be related to iatrogenic inflammation of the skin: Haro et al. [24] found that a patient previously treated with radiotherapy for breast cancer later developed LyP in the treated area.

**Figure 1:**
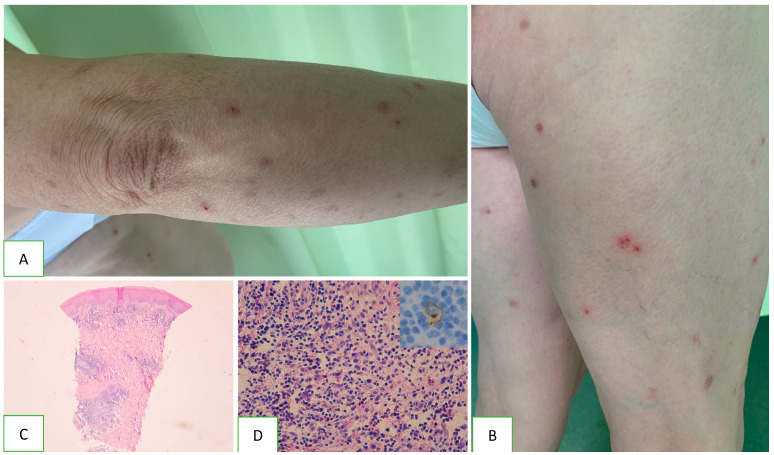
Clinical characteristics and histopathology of lymphoid papulosis. Scattered papules and single nodules are visible on the skin of the limbs and trunk (**A**). Some lesions have a haemorrhagic component, and others present disintegrations in the centre (**B**). Typical arrangement of the cutaneous infiltrate with nodular aggregates sparing the epidermis (**C**). Cellular composition contains small lymphocytes and scattered eosinophils with single atypical CD30+ cells (**D**).

Lymphomatoid papulosis manifests as polymorphic, varicella-like papules, sometimes vesicles, necrotic, ulcerated or haemorrhagic lesions [21] (Figure 1). They are most common on the limbs and trunk; however, in some cases, they are also found on the genitals and oral cavity. The lesions may be accompanied by itching or slight tenderness or may be asymptomatic. The main feature of Lyp that distinguishes it from other types of CD30+ LPD is that the lesions spontaneously resolve within four to eight weeks, in which case, resolution may be permanent or a few years [25]. The disorder occurs in adults, with a slight predominance of men aged 20 to 40 years, but paediatric cases have also been reported [21]. The WHO-EORTS 2018 update classifies LyP into six subtypes, viz. LyP A, B, C, D, E and LyP with DUSP22-IRF4 rearrangement; these differ slightly in histopathology and immunophenotypes, but some features overlap [13].

The most common subtype is LyP A > 80%. All subtypes have an indolent clinical course and similar clinical presentation, with the exception of subtype E; this subtype manifests as vasculitis involving small and medium vessels with large ulcerative lesions [26]. The immunophenotype of the LyP cells varies by subtype. They are generally represented by CD4+CD8− or CD4−CD8+, but CD4+CD8+ cases are rare. LyP with *DUSP22* is more commonly CD4−/CD8−. Variable loss of pan T cell antigens (CD2, CD3, CD5 and CD7) can be observed. Most cases are CD30+, BF1+, granzyme B+ and CD56+/−. Clonal rearrangement of T-cell receptor (TCR) genes has been reported in more than 50% of cases of LyP. The majority of LyP cases express the alpha/beta TCR. However, expression of the gamma/delta TCR has been observed in type D [15].

The diagnosis of LyP is based on a combination of clinical features and histopathological and immunohistochemical findings [21]. It is necessary to perform a complete blood count, LDH determination and basic blood biochemistry. If a secondary malignancy is suspected, e.g., the presence of enlarged lymph nodes, B symptoms or skin lesions not resolving spontaneously, the diagnostics should be extended to include further laboratory and imaging tests, possibly in combination with a diagnostic with further laboratory and imaging tests, and maybe with bone marrow biopsy [1].

### 2.2. Primary Cutaneous Anaplastic Large Cell Lymphoma

**Figure 2:**
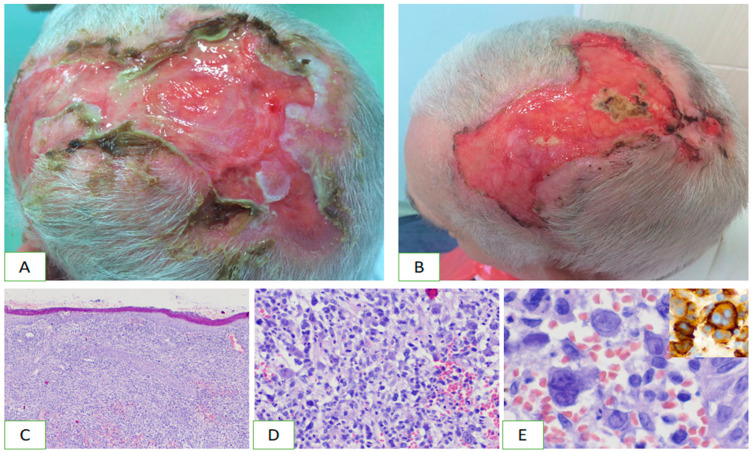
Clinical characteristics and histopathology of primary cutaneous anaplastic large cell lymphoma. On the top of the head, there are extensive merging ulcers accompanied by pain (**A**,**B**). Diffuse infiltration of lymphoma cells involves the skin (**C**). It contains atypical pleomorphic cells (**D**). Mononucleated and multinucleated cells prevail and show strong CD30 expression (**E**).

Primary cutaneous anaplastic CD30+ cell lymphoma belongs to the subgroup of peripheral T-cell lymphomas (PTCL). It was described in 1985 as a CD30-expressing malignancy of the lymphatic system, which, in most cases, affects only the skin [27,28]. It contains large cells with pleomorphic, immunoblastic or anaplastic morphology (Figure 2). The CD30 antigen can be found in at least 75% of neoplastic cells [19,29]. The condition accounts for 9% of all CTCLs. It usually affects the elderly (median 60 years), with a greater prevalence among men, but has also been reported in children [18]. The 10-year survival rate for pcALCL is 95%, while the 5-year survival rate is 93% for ALK-positive ALCL and 37% for ALK-negative ALCL [25,30].

Primary cutaneous anaplastic large cell lymphoma is clinically manifested as solitary or multiple nodules, tumours, erythematous plaques and sometimes papular lesions, often accompanied by ulceration (Figure 2). They tend to grow bigger over weeks and usually involve the head, neck and limbs. Extensive limb disease (ELD), a variant of pcALCL, is manifested by multiple skin tumours on one limb and is associated with disease progression and poorer prognosis [31]. Additionally, pcALCL is less likely to demonstrate spontaneous regression than LyP, ranging from 10% to 42% of patients, and is often subject to recurrences [32]. Extracutaneous involvement is rare and most commonly involves regional lymph nodes, which are observed in 10% of patients [25]. The secondary involvement of the skin by systemic ALCL, HL and MF CD30+ should be excluded.

The classic histological picture presents limited cellular infiltration and large lymphoid cells, usually with absent or discrete epidermotropism. The immunophenotype includes CD4+, CD30+, BF1+, CD56+/−, granzyme B+ cells, and variable loss of CD2, CD3 and CD5. Unlike nodal ALCLs, most pcALCLs express CLA cutaneous lymphocyte-associated antigen) but do not express EMA (epithelial membrane (antigen) [33,34].

As in the case of LyP, the diagnosis of pcALCL is based on a combination of clinical features and histopathological and immunopathological findings. Basic laboratory tests are necessary, as in LyP; however, these should be accompanied by imaging of the whole body, preferably with PET-CT. Additionally, it is worth testing for HIV, HTLV-1 and EBV because some T-cell lymphomas may produce secondary skin lesions and have a viral aetiology. If the lymph nodes are enlarged or metabolically active in PET, a lymph node biopsy should be performed. Bone marrow biopsy is no longer recommended in patients with pcALCL unless the patient has systemic symptoms, cytopenia or extensive skin lesions [15,18].

## 3. Genetics of Primary Cutaneous CD30-Positive Lymphoproliferative Disorders

Molecular tests ease the diagnosis of cutaneous CD30+ lymphoproliferative disorders and help differentiate LyP and pcALCL. It is also important to differentiate pcALCL and secondary skin involvement by anaplastic lymphoma kinase (ALK)-positive and ALK-negative ALCL: the latter requires different treatment methods and entails a worse prognosis. ALK is a tyrosine kinase receptor. Its physiological expression is limited to a few cell types, such as endothelial cells or glial cells. ALK fusion has been noted in pcALCL but not LyP. Until recently, pcALCL has been considered an ALK-negative lymphoma, although pcALCL may also be ALK-positive [35]; this is a rare phenomenon which occurs practically only in the paediatric population and is associated with progression to the secondary systemic involvement [36].

A new LyP subtype was recently identified, characterised by a rearrangement of DUSP22 and IRF4 (Interferon Regulatory Factor 4), a tumour suppressor gene regulating T-cell proliferation, at the 6p25.3 locus; the same rearrangements have been previously reported in pcALCL, where they are observed in 20–57% of cases [37]. DUSP22—IRF4 gene rearrangement has also been described in systemic ALCL and transformed MF [38].

Another study identified chromosomal translocations targeting CD30+ LPD tyrosine kinases. Among the 47 patients with LPD, 4% carried the NPM1 (5q35) and TYK2 (19p13) fusion, which encodes the NPM1-TYK2 protein. This protein promotes cell proliferation by activating the STAT1/3/5 pathway; this information may be valuable when using tyrosine kinase inhibitors in patients carrying this fusion [35].

Sun et al. found that in most LPDs, the SATB1 protein (binding AT1-rich sequences), i.e., a nuclear protein of thymocytes that plays a role in T cell development, is overexpressed in CD30+ lymphocytes [39]. Overexpression has been noted in 91.7% of LyP and 38.1% of pcALCL cases, with the prevalence increasing as the disease progresses. Interestingly, these cases have a better response to methotrexate (MTX).

Epigenetics can also be used to differentiate between CD30 neoplastic proliferation and CD30+ inflammatory infiltrates. De Souza et al. evaluated the expression of intracellular 5-hydroxymethylcytosine (5-hmC) caused by DNA cytosine methylation at position 5 [40]. This phenomenon occurs in several malignancies. The authors report that 5-hmC expression occurred in 27 LyP and 14 pcALCL cases and in 19 CD30+ inflammatory infiltrates. In contrast, a complete loss of 5-hmC expression was observed in 63% of LyP and 53% of pcALCL cases; such a lack of expression was a hallmark of CD30+ LPD, and this may help to distinguish neoplastic diseases from CD30+ inflammatory infiltrates [40].

Finally, Kamstrup et al. demonstrated Notch expression in 12 LyP and in 11 pcALCL cases; the transmembrane Notch receptor influences T lymphocyte proliferation and demonstrates strong expression in pcALCL lymphocytes but lower in LyP cells [41]. This discovery may determine the future use of targeted therapy with Notch antagonists and may help to differentiate LyP from pcALCL.

## 4. Pharmacology of Brentuximab Vedotin

**Figure 3:**
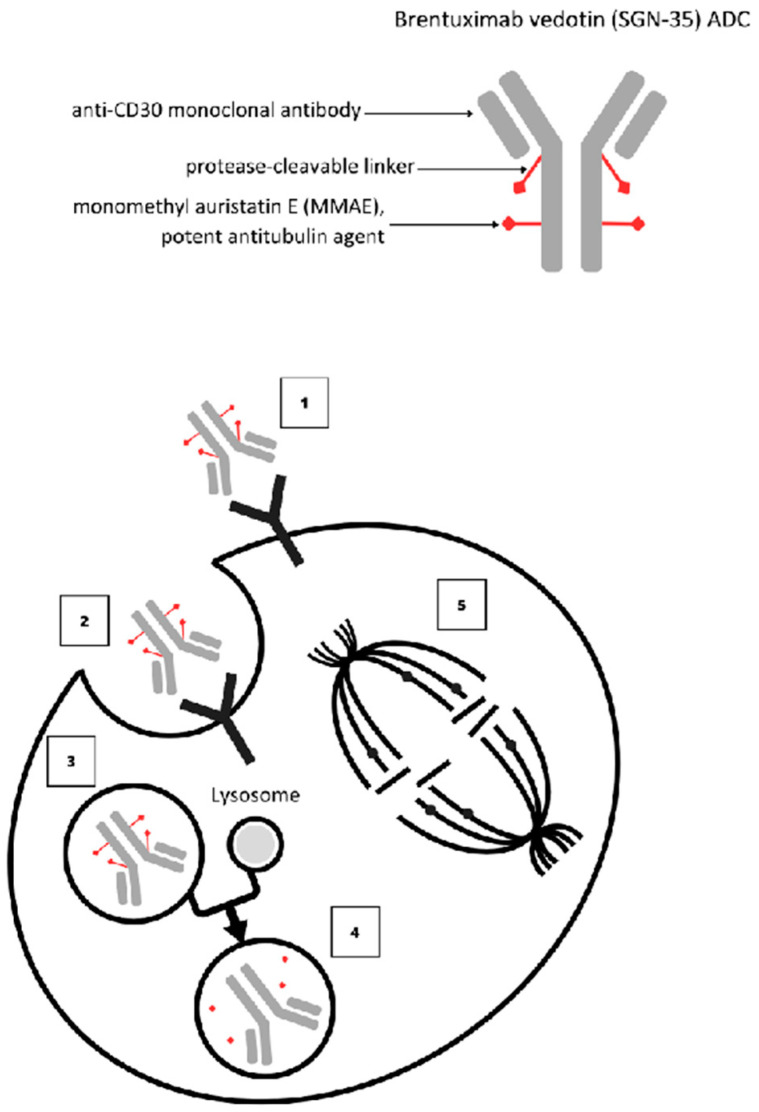
Brentuximab vedotin—mechanism of action. ADC binds to CD30-ADC-CD30-complex (1). Endocytosis (2). ADC-CD30 complex traffics to lysosome (3). MMAE released by lysosomal proteases (4). MMAE binds to tubulin and disrupts microtubule network, leading to G2/M cell cycle arrest and apoptosis (5).

Brentuximab vedotin is a conjugate of a mouse–human chimeric IgG1 anti-CD30 antibody linked to monomethyl auristatin E (MMAE), which is an anti-tubulin drug. MMAE is a synthetic drug based on the auristatin structure. It inhibits microtubules by promoting the breakdown of these structures and inhibiting tubulin polymerisation. After entering the body, the conjugate binds selectively to CD30 on the surface of LPD30+ cells. It then enters the cell by endocytosis and joins the lysosomes as a vesicle, where free MMAE is released by proteolytic enzymes. This inhibits the polymerisation of tubulin in the cellular cytoskeleton, arrests the G2/M cell cycle and leads to apoptosis of LPD30+ cells (Figure 3) [42]. MMAE can induce the death of neighbouring cells by diffusion across the cell membrane [43]. Brentuximab vedotin was first approved for the treatment of classic HL and ALCL in the US in 2011 and in Europe in 2012. According to the NCCN 2021 recommendations, BV is a basic drug for the treatment of pcALCL and a first- or second-line drug in the case of CD30+ MF [44]. In accordance with the European Medicines Agency (EMA), BV is indicated for adult patients with previously untreated CD30+ Stage III or IV HL in combination with doxorubicin, vinblastine and dacarbazine (AVD); it is also available for the treatment of adult patients with CD30+ HL who are at increased risk of relapse or progression following autologous stem cell transplant and for adult patients with relapsed or refractory CD30+ HL. In addition to HL, BV is indicated for the treatment of systemic anaplastic large cell lymphoma in combination with cyclophosphamide, doxorubicin and prednisone (CHP). BV is also indicated for the treatment of adult patients with CTCL CD30+ after at least one prior systemic therapy [45]. As part of the drug program (B66) in Poland, BV is indicated in patients with histopathologically confirmed CTCL, viz. pcALCL and MF. However, this program requires the CD30 antigen to be confirmed immunohistochemically in at least one of the biopsies.

Previous studies have also examined the interaction of BV with ketoconazole, rifampicin, midazolam and cytostatic drugs. The co-administration of BV with rifampicin, a strong CYP3A4 inducer, did not alter the plasma exposure to BV and appeared to reduce plasma concentrations of detectable metabolites of MMAE, an antimicrotubule agent. BV co-treatment did not alter the metabolism of midazolam, a CYP3A4 substrate. In addition, the co-administration of BV with ketoconazole, a strong P-gp and CYP3A4 inhibitor, increased plasma exposure to MMAE by approximately 73% but had no effect on exposure to BV. As such, it appears that the co-administration of BV with strong CYP3A4 and P-gp inhibitors may increase the incidence of neutropenia. Finally, BV treatment did not affect plasma exposure to AVD, nor is it expected to affect exposure to CHP [45].

Jagadeesh et al. report the efficacy of BV to be independent of the percentage of CD30 cells. The authors analysed five studies encompassing a total of 275 patients with PTCL, CTCL and B-cell non-Hodgkin lymphoma (NHL) [46]. In 140 patients, tumours presented CD30 expression < 10%; among these, 60 patients demonstrated CD30 expression undetectable by immunohistochemistry. No significant differences in overall response rates (ORR) were observed between patients with CD30 expression > 10% and those with <10% in any of the studies. The median duration of the response was also similar. Therefore, CD30 expression, as measured by standard immunohistochemistry, does not appear to influence the clinical benefits of using BV.

As BV treatment may result in tumour lysis syndrome (TLS), appropriate prophylactic treatment should be applied, including a pre-treatment determination of uric acid level and the application of prednisone, allopurinol and hydration. In patients with advanced disease, rasburicase may be considered, as well as hospitalisation for the first administration of the drug, with subsequent observation over several days. In addition, blood tests should be performed one week after administration in order to monitor possible bone marrow damage. Premedication of an allergic reaction should also be considered, especially before the first administration of the drug. Contraception is required during BV treatment. Additionally, a man should consider semen analysis prior to treatment [45].

Brentuximab vedotin is generally well tolerated; however, side effects are frequent and present a challenge for haematologists and dermatologists. In order of frequency, they most often include infections, followed by peripheral sensory neuropathy, nausea, fatigue, diarrhoea, pyrexia, upper respiratory tract infection and neutropenia. One of the most serious, but also the rarest, side effects is the reactivation of the John Cunningham (JC) virus, which causes progressive multifocal leukoencephalopathy (PML). PML is a demyelinating disease of the CNS that is often fatal [45]. Most data related to the side effects of BV have been described in patients suffering from HL.

One of the biggest concerns associated with BV treatment is peripheral neurotoxicity, which is observed to some degree in most patients receiving BV; this is the main reason for dose modifications, delays in administration or discontinuation of treatment and may affect the efficiency of the therapy. Peripheral neuropathy can cause sensory, motor and autonomic dysfunctions of the peripheral nerves. Importantly, peripheral neuropathy is largely reversible but may persist for months or years after treatment. Therefore, it may become a major survival problem for patients treated with the drug [47]. The mechanism of developing neuropathy during BV therapy is related to the inhibition of microtubule-dependent axonal transport. Microtubules maintain axonal transport between nerve cell bodies and distal nerve endings [48]. Axonal degeneration typically results in neuropathy of the most peripheral nerve endings, starting at the most distal parts of the extremities, such as the fingertips and pulp of the toes; however, this condition later progresses proximally towards the trunk. Of note, peripheral neuropathy has been associated with other MMAE antibody conjugates, such as enfortumab–vedotin (Padcev) used in the treatment of metastatic urothelial carcinoma and/or polatuzumab–vedotin (Polivy) used in diffuse large B-cell lymphoma (DLBCL) [49,50]. Interestingly, neurons do not express CD30, as confirmed by the pathological data from a sural nerve biopsy of a patient with BV-induced peripheral neuropathy [51]. Although the route by which MMAE reaches the peripheral nerves remains unclear, several potential mechanisms exist. Firstly, monomethylauristin E can diffuse from CD30-positive lymphoma cells into the extracellular matrix and kill the surrounding CD30-negative cells, although the extent of MMAE action outside the tumour environment is unknown [52]. Also, the drug can be released before being internalised with CD30+ cells, thus damaging other cells [53]. Although small molecules, such as MMAE, can enter cells by passive diffusion and be transported within peripheral nerve cells, systemic levels of unconjugated MMAE are typically very low and are hence unlikely to damage the peripheral nerves [54].

Brentuximab vedotin may cause motor, sensory and autonomic nerve dysfunction. Patients most often complain of sensory symptoms, including abnormal vibratory sensation (80%), abnormal tactile perception (80%), paresthesia (70%), numbness (70%), tingling (60%) and burning (40%). They may also report allodynia, i.e., pain in response to usually painless stimuli, or hyperalgesia, i.e., excessive pain in response to painful stimuli. Distal loss of vibratory sensation is most readily detected on physical examination [55].

The level of exposure to MMAE and its duration in the peripheral nerve tissue is believed to most strongly influence peripheral neuropathy. More severe neuropathy was associated with more cycles of BV and more frequent dosing [54]. It was found that 22% of patients experienced peripheral neuropathy at a dose of 1.8 mg/kg, given every three weeks [56], compared to 66% at a weekly dose of 0.4–1.4 mg/kg [57]. This may be due to the repair mechanisms having insufficient time to act.

In the ALCANZA study, 44 of the 66 (67%) patients from the BV group experienced peripheral neuropathy: 18 patients demonstrated Grade 1 neuropathy, 20 had Grade 2 and 6 had Grade 3; no cases of Grade 4 were noted. Of the 44 patients, 23 (52%) required treatment modifications, e.g., dose reduction, dose interruption or infusion delay, while 9 (14%) permanently discontinued BV treatment. In the final data, 26 of the 44 patients experienced complete resolution of peripheral neuropathy, while 12 exhibited an improvement to Grade 1 or 2 [58]. Patients treated with BV should receive multidisciplinary care. In such cases, a neurologist can quickly detect even discreet symptoms of peripheral neuropathy via physical examination. The first symptom to appear seems to be loss of vibratory sensation, which can be assessed with a tuning fork [51,55]. Most available literature on the treatment of peripheral neuropathy is based on patients with HL. Pharmacological treatment of peripheral neuropathy is focused mainly on the symptomatic approach, mainly using duloxetine. This drug inhibits serotonin and norepinephrine reuptake and is recommended for treating BV-induced peripheral neuropathy by the American Society of Clinical Oncology (ASCO) clinical practice guidelines. Duloxetine relieves numbness and tingling and is effective in reducing pain. Apart from duloxetine, other drugs have also been used, such as tricyclic antidepressants, gabapentin, and baclofen, but despite a good response, no guidelines have been created for the treatment of neuropathy caused by BV. Improvements in motor strength were also observed in some patients after drug residue plasma exchange sessions. In addition to pharmacology, physiotherapy may be a helpful intervention. Brentuximab vedotin infusions may result in disabilities, including sensory and movement disorders. While studies suggest that physical activity, such as sensorimotor training and balance training, can potentially reduce the severity of existing neuropathy, the current body of evidence is unfortunately insufficient to support this approach [47].

## 5. Standard Treatment

### 5.1. Lymphomatoid Papulosis

The best-documented therapeutic approach to LyP involves the use of topical glucocorticoids, methotrexate (MTX) and phototherapy (UVA and UVB). Nevertheless, the initial watch-and-see strategy may be the first-line treatment due to the mild and self-limiting nature of the disease. Importantly, there is no evidence that early treatment prevents the development of a second malignancy or affects the course of the disease [59]. A large retrospective, multicentre study showed no difference in the final CR (complete response) in patients treated with one of the options: topical corticosteroids, methotrexate or phototherapy [60]. Both UVB and PUVA therapies have been successfully used in the treatment of LyP. Generally, PUVA is superior to UVB nb311; however, due to the recurrent nature of LyP and the limited lifetime dose of PUVA, UVB nb311 is usually the first-line treatment [61]. Kempf et al. [27] reported 27% CR and 68% PR among 19 patients treated with PUVA. In most cases, phototherapy treatment needs to be restarted when the disease recurs.

One effective treatment option in LyP is MTX; however, discontinuation is associated with a relapse rate of approximately 40%, and long-term therapy is needed. Fernandez-De Misa et al. studied 48 patients treated with <20 mg MTX per week, resulting in a CR in 25 patients, a PR in 21 patients, and a non-response in 2 [60]. Among the patients with a CR, 92% experienced a LyP relapse after discontinuation of MTX therapy. Vonderheid et al. studied 45 patients treated with MTX at a median dose of 20 mg/week for 39 months [62]. In most cases, response to treatment was observed within four weeks, and long-term disease control was reported in 39 patients. After achieving a response to methotrexate, the frequency of methotrexate dosing was reduced to as low as 1 per month. After methotrexate discontinuation, 10 patients remained LyP-free for a median follow-up of 127 months.

Although methotrexate therapy is a long-term option, it has been found that reducing the dosing to a weekly dose after remission of skin lesions is effective. Other ways of treating LyP have been described, such as local radiotherapy, interferon-alpha, bexarotene, multidrug chemotherapy and mycophenolic acid derivatives. However, these methods are less common or have not been adopted at all in everyday medical practice.

One of the newer treatments of refractory, extensive cases is CD30-targeted therapy with BV. Lewis et al. used BV in 12 patients with LyP, all of whom received 1.8 mg/kg brentuximab vedotin infused over 30 min every 21 days [63]. All the patients responded to BV, and seven showed a complete response. For all patients, the time to respond was three weeks. The median duration of response was 20 weeks (between 6 and 103 weeks). In five patients, a relapse was reported with a median time to relapse of 12 weeks (between 6 and 41 weeks). One patient with relapse was re-treated and remained in a partial response for more than 23 months.

### 5.2. Primary Cutaneous Anaplastic Large Cell Lymphoma

The therapeutic strategy for pcALCL is different from that of LyP. In the case of single lesions, the preferred approach consists of local radiotherapy and surgical excision of the lesions. In multifocal pcALCL, systemic therapy is applied. The literature also describes new possibilities for targeted therapies based on molecular data. Single lesions can be surgically removed or subjected to local radiotherapy at 30–40 Gy [64,65]. In most cases, these are first-line therapies. Importantly, the two treatment methods are not used in combination, as this has not been found to yield any further benefits. While such management leads to remission, it typically results in relapse that requires additional therapy.

In the case of multifocal lesions, MTX is the treatment of choice. Due to the long-term nature of the therapy and the need to reduce toxicity, it is used in the lowest possible weekly dose, allowing for remission. Once the disease is in remission, the frequency of administration can be reduced. A large retrospective study showed a 77% response rate within four weeks, although ongoing therapy is required to prevent the recurrence of skin lesions [62].

In addition, the NCCN 2021 guidelines recommend bexarotene for multifocal lesions in pcALCL. It is an oral drug, a vitamin A derivative that binds to the X receptor. However, as noted in the guidelines, the data on its use in pcALCL has been obtained from limited case reports, and it has yet to be accepted into everyday medical practice [44].

Currently, the favoured treatment for cases of extracutaneous involvement in pcALCL, characterised by the N1 peripheral lymph nodes, is multidrug chemotherapy based on the CHP (cyclophosphamide, doxorubicin, prednisone) or CHOP (CHP + vincristine) regimen. However, the preferred regimen for N1 cALCL is BV with or without local radiotherapy [44,66,67,68,69,70,71,72]. Duvic et al. presented the results of the treatment with brentuximab vedotin conducted in 48 patients, of which 11 were diagnosed with pcALCL or LyP (2 with pcALCL, 9 with LyP), the remaining patients suffered from MF CD30+, LyP/MF, pcALCL/LyP or pcALCL/MF [67]. All patients received intravenous BV 1.8 mg/kg every three weeks. Interestingly, skin lesions in LyP and pcALCL responded faster to the treatment than the lesions in MF but demonstrated a shorter response. The time to respond in LyP/pcALCL was 3 weeks (range: 3 to 9 weeks) compared to 12 weeks (range: 3 to 39 weeks) in patients with MF. The median duration of response was 26 weeks (range: 6 to 44 weeks) in LyP/pcALCL patients compared to 32 weeks (range: 3 to 93 weeks) in patients with MF. The objective response rate (ORR) for all patients was 100%.

Muniesa et al. analysed 67 patients with CTCL who were treated with BV from the Spanish Registry of Cutaneous Lymphomas (RELCP) [68]. The study included 12 patients with CD30 lymphoproliferative disorders, 2 with LyP and 10 with pcALCL. Eight patients with pcALCL presented with extracutaneous involvement. The patients received a median of seven BV infusions, and the mean follow-up period was 18 months. Most patients in this group showed a rapid and durable response to BV with a median progression-free survival (PFS) of 23,2 months. In patients with LPD CD30+, the ORR was 84%, and the CR rate was 42%. These results confirm the effectiveness of BV treatment.

The largest international, randomised, multicentre phase III study (ALCANZA) compared the effects of the treatment with BV 1.8 mg/kg, administered every three weeks, for up to 16 cycles with bexarotene 300 mg/m^2^ given daily for up to 48 weeks and oral methotrexate 5–50 mg administered weekly for up to 48 weeks [58,69]. The study groups comprised 128 previously treated patients with CD30+ MF (97 patients) and pcALCL (31 patients). The patients were randomly assigned to each group: 16 patients with pcALCL received brentuximab vedotin, and 15 patients were given bexarotene or methotrexate. The primary endpoint was the proportion of patients who achieved an *ORR* lasting at least four months (ORR4). The ORR4 values were 69% (*n* = 11) in the BV group compared to 20% (*n* = 3) in the bexarotene/methotrexate group. Importantly, PFS was 22.2 months longer for BV compared to bexarotene/MTX (i.e., 27.5 vs. 5.3 months) [58,69]. Based on the findings, BV was approved at a dose of 1.8 mg/kg for the treatment of pcALCL and MF CD30+.

In a subsequent study, Ribereau-Gayon et al. evaluated the effectiveness of treatment for aggressive CD30+ CTCL [70]. From April 2015 to January 2022, seven patients were included in the study: four with MF with large cell transformation, one with primary cutaneous aggressive epidermotropic CD8-positive T-cell lymphoma and two with pcALCL. The patients with pcALCL outside the skin had lymph node involvement. They had no B symptoms or elevated LDH levels. In all biopsies, the anaplastic cells showed strong CD30 positivity. The aim of the study was to determine the effectiveness of treatment with a combination of CHP with BV. Patients received six cycles of BV+ CHP administered at three-week intervals: BV was given at 1.8 mg/kg, cyclophosphamide at 750 mg/m^2^, doxorubicin at 50 mg/m^2^ intravenously on day 1 of each cycle; prednisone was given at a dose of 100 mg, once daily, orally, on days 1 to 5. Additionally, all patients received granulocyte colony-stimulating factor (G-CSF) and had regular skin and lymph node examinations. The first PET-CT evaluation was performed after six cycles [70]. The follow-up period was 16.4 months in one patient and 9.6 months in the other. Both subjects achieved ORR4. PFS was 16.4 months in the first patient and 9.6 months in the second. Both patients achieved CR, with no progression or recurrence observed at the end of the study. The pcALCL patients responded more quickly to treatment, which is consistent with previous studies of BV monotherapy; however, this was the first real-world cohort study to be performed; the findings indicate BV has a favourable safety profile and good effect. It also appears to be an alternative instead of CHOP for aggressive treatment of CD30+ CTCL in cases of extracutaneous involvement, but it requires further study.

Milan et al. report the case of a patient with pcALCL who was treated with a short course of BV [71]. The patient achieved a complete remission of the disease after two cycles of treatment. In cases of pcALCL, CD30 is expressed in>75% of cells; therefore, approximately 75% of cells should die after each cycle of BV. The patient showed a significant reduction in lesion size, consistent with the MMAE pharmacokinetics, which peaks at one to three days after infusion.

Guarnera et al. describe the case of a 45-year-old patient with extracutaneous, recurrent and aggressive pcALCL. The patient was treated with autologous stem cell transplantation (ASCT) with consolidation with BV [72]. BV was administered at a dose of 1.8 mg/kg every 21 days for six cycles, resulting in complete remission of the disease. The patient subsequently underwent ASCT and a further 10 cycles of BV as part of consolidation therapy, for a total of 16 cycles. Thirty months after the last dose of BV, the patient remained in complete remission. Larger clinical studies are summarised in Table 1.

## 6. Perspectives

Currently, the recommended first-line therapies for treating localised primary cutaneous CD30-positive lymphoproliferative disorders are complete surgical excision and local radiotherapy. However, the most appropriate treatment for generalised and relapsed/refractory patients remains more controversial. In recent years, BV has been indicated as one of the best options for achieving high response rates with low toxicity; however, new molecular-based therapies are still being sought for these patients [19,73]. One option is based on the use of Janus JAKs, a family of non-receptor tyrosine kinases involved in the intracellular signal transduction of the JAK-STAT pathway [74]. The signal cascade influences the life processes in the cell. STAT3 or JAK1 mutations are associated with pSTAT3 in pcALCL, with NPM1-TYK2 gene fusion and oncogenic activation of STAT3. Upon activation by cytokines, it is phosphorylated and translocated to the nucleus, where it acts as a transcription activator, affecting many cellular processes, including proliferative and anti-apoptotic effects. In vitro studies have found inhibitors of these kinases to potentially be effective in controlling pcALCL cells [75,76].

NOTCH-1 is a transmembrane protein that controls the further fate of the cell by playing a role in developmental processes [75,76]. It is a signalling network that regulates interactions between neighbouring cells. The release of NOTCH1 (ICN1) from the membrane-associated protein NOTCH1 is prevented by gamma-secretase (γ-secretase) inhibitors. The resulting cascade downregulates the NFkB pathway of tumour cells, leading to the inactivation of survival genes.

Some types of haematological malignancies, including ALCL, are driven by the anaplastic lymphoma kinase (ALK) gene [77]. A variety of ALK inhibitors are available, including crizotinib, ceritinib, lorlatinib, alectinib, entrectinib and brigatinib, that can downregulate the STAT3 pathway in patients with ALK-positive pcALCL, thereby causing apoptosis among tumour cells [78].

Other agents with possible activity in pcALCL LyP include Denileukin Difitox (DD) [79,80,81]. Denileukin Difitox is a chimeric immunotoxin consisting of interleukin 2 (IL-2) and the cytotoxic domain of diphtheria toxin. It acts on cells that express the interleukin-2 receptor, such as activated T cells in cutaneous T-cell lymphomas. After binding to the receptor, it undergoes endocytosis and releases a diphtheria toxin that can inhibit protein synthesis, resulting in cell apoptosis [79]. Denileukin Difitox was approved by the U.S. Food and Drug Administration in 1999 for the treatment of relapsing CTCL.

Both pcALCL and LyP are characterised by high expression of CD30, and as such, may be a suitable target for chimeric antigen receptor T-cell (CAR-T) therapy [82]. Preclinical studies demonstrated that CD30 CAR-T cells lyse MyLa CTCL cells and inhibit tumour growth [83]. Also, a study of nine patients with CD30+ lymphoma found treatment based on a CD30 CAR-T infusion to be well tolerated with no immunodeficiency noted [84]. Although many studies have evaluated the effectiveness of CD30 CAR-T, especially for the treatment of HL, no studies have yet been performed on CTCL. Importantly, both pcALCL and LyP demonstrate higher CD30 expression than MF and SS, and CAR-T therapy would hypothetically be more effective in tumours with high CD30 expression.

## 7. Conclusions

Long-term observation has shown that BV-targeted therapy is effective in LPD30+, especially in pcALCL. In our opinion, BV should not be used in patients with lymphomatoid papulosis; the condition has a benign and self-limited course, and the risk of side effects resulting in lower quality of life, particularly peripheral neuropathy, is higher than the benefits of treatment. Future research should focus on developing alternative regimens, including longer intervals between infusions, fewer cycles, or administering BV directly to single lesions. In addition, there is a need to develop novel treatment regimens that can reduce the incidence of side effects without affecting the final treatment responses; among these, bispecific antibodies and cellular therapies such as anti-CD30 CAR-T cells appear particularly most promising.

## Figures and Tables

**Table 1:** Larger clinical trials of brentuximab vedotin in primary cutaneous CD30-positive lymphoproliferative disorders.

Authors/Reference	Disease and Number of Patients	Treatment	Prior Systemic Therapies	Response	Median Response Duration (Range)	OS	Adverse Events
Duvic et al. 2015 [67]	Total 48, MF 28, pcALCL 2, LyP 9, LyP and MF 7, ALCL/LyP/MF 2	BV at 1.8 mg/kg/iv every 21 days for a maximum of eight doses.	Median 2 (1–10) for MF, and 1 (0–5) for LyP/pcALCL	Total OR—73%, CR—35%, MF OR 54%, ALCL/LyP/MF OR 100%	LyP/pcALCL 26 weeks (6–44), MF—32 weeks (3 to 93)	14.7 years (10.2—not reached) from 1st diagnosis	Peripheral neuropathy (67%), nausea (19%), fatigue (35%), rash (24%), diarrhoea (15%), myalgia (17%), localised skin infection (15%), neutropenia (15%), alopecia (11%)
Lewis et al. 2017 [63]	LyP—12	BV 1.8 mg/kg every 21 days for a maximum of 8 doses	GKS—10 MTX—4 UV-B—3	OR—100%, CR 58%	20 weeks (6–103 weeks)	NR	Peripheral neuropathy 83%, fatigue 58%, nausea 50%, diarrhoea 42%, hypersensitivity drug rash 42%, headache 33%, neutropenia 17%, abdominal pain 17%, vomiting 17%
Horwitz et al. 2021 [58]	pcALCL—64	BV 1.8 mg/kg every 3 weeks for up to 16 cycles	At least 1 prior systemic therapy or radiotherapy	OR 54.7%, CR 17.2%	16.7 m	3-yrs OS—64.4%	Peripheral neuropathy —67%, nausea—36%,diarrhoea—29%, fatigue—29%, vomiting—17%, alopecia—15%, pruritus—17%, pyrexia—17%
Muniesa et al. 2023 [68]	Total 67, MF—48, SS—7, pcALCL—10, LyP—2	BV 1.8 mg/kg iv every 3 weeks	Mean previous systemic treatments—of 4 (1–11)	Total OR 67%, CR 37%; pcALCL and LyP OR 84%, CR 42%	Total 10.3 m, pcALCL and LyP 23.2 m	1 death due to disease progression	Peripheral neuropathy 57%. Serious AE: Skin rash—2, local skin infection—2, sepsis—1, inflammatory adenopathy 1, pyoderma gangrenosum 1, palmoplantar pompholyx 1, anaemia+/− thrombopenia 1, hepatitis —1, pancreatitis—1, acute renal failure—1, asthenia—1.
Ribereau-Gayon et al. 2023 [70]	Total 7, MF—4, pcALCL—2, aggressive epidermotropic T-cell lymphoma—1	BV + CHP administered at 3-week intervals *	0–2 pts, 1–3 pts, 2–1 pt, 3–1 pt.	OR 6/7 (86%) CR—3 PR—3	14.9 m (11.6–16.4)	No data	Anaemia—6, diarrhoea—5, fatigue—5, pruritus—4, nausea—3, bone pain—3, thrombopenia—3, and neutropenia—3.

* BV 1.8 mg/kg, cyclophosphamide 750 mg/m^2^, doxorubicin 50 mg.m^2^ iv on day 1 of each cycle, and prednisone at 100 mg once daily orally, days 1 to 5 of each cycle. Abbreviations: ALCL—anaplastic large cell lymphoma; LyP—lymphomatoid papulosis; pc—primary cutaneous, BV—brentuximab vedotin, CHP—cyclophosphamide, doxorubicin, prednisone; CR—complete response, GKS—steroids, LyP—lymphoid papulosis, m—months, pcALCL—primary cutaneous anaplastic large cell lymphoma, MF—mycosis fungoides, MTX—methotrexate, NR—not reported, OR—overall response, PR—partial response.
